# Effect of Earthworm Digestion on Abundance, Composition and Diversity of Bacterial Pathogens in Sewage Sludge from Wastewater Treatment Plants

**DOI:** 10.3390/microorganisms13112507

**Published:** 2025-10-31

**Authors:** Manuel Aira, Jorge Domínguez

**Affiliations:** Grupo de Ecoloxía Animal (GEA), Universidade de Vigo, E-36310 Vigo, Spain; jdguez@uvigo.gal

**Keywords:** earthworms, bacterial diversity, bacterial human pathogens, sewage sludge, vermicomposting

## Abstract

The increased production of sewage sludge is a major environmental concern as the sludge contains hazardous components, particularly human bacterial pathogens (HBPs). Transit of sewage sludge through the earthworm gut reduce or even eliminate HBPs and modify bacterial taxonomic and functional composition. However, it is unclear whether the effect is general or dependent on the type of sewage sludge involved. We characterized the taxonomic and functional profiles of bacterial assemblages in sewage sludge from different wastewater treatment plants (WWTPs), before (sludge) and after earthworm gut transit (casts). We found that composition and diversity of both taxonomic and functional bacterial communities of sludge and casts were significantly different. However, these differences varied among WWTPs with both increases and decreases in composition and diversity after gut transit. Interestingly, most bacterial taxa present in earthworm casts were not detected in the original sewage sludge. All sludge samples initially contained low levels of HBPs, which were significantly reduced or eliminated in earthworm casts. Nevertheless, gut transit increased the abundance of some HBPs. Further studies should determine whether vermicomposting effectively eliminates these HBPs and whether the differences in earthworm cast bacterial communities, which are dependent on the sewage sludge source, persist in the final vermicompost.

## 1. Introduction

The growing need to treat polluted urban water has led to a continual increase in the production of sewage sludge worldwide [1]. Despite improvements in wastewater treatment [2], the sludge contains high levels of various pollutants, including pharmaceutical residues, heavy metals and pathogens [3]. Overall, this poses a significant health risk, particularly since land application remains the primary method of disposing of sewage sludge, despite concerns regarding wastewater treatment [4]. Among the pollutants contained in sewage sludge, human bacterial pathogens (HBPs) are of special concern, because of their virulence, abundance and diversity [5] and also because, given the high loads of antibiotics present in wastewater, they may acquire or develop antibiotic resistance genes [6,7]. This concern is heightened by the persistence of most pathogens and resistance genes even after conventional wastewater treatment [8], eventually leading to an increase in the pathogens/genes in soil [9].

Vermicomposting biotechnology, which makes use of the combined action of microorganisms and earthworms, is capable of converting sewage sludge into a valued organic amendment [10]. Vermicomposting can reduce microbial pathogen loads [11,12] and also the numbers of antibiotic resistance genes [13]. Vermicomposting involves two main steps. The first step involves the digestion of substrates by earthworms, which produces earthworm casts. In the second step, the casts undergo a maturation process, yielding the final vermicompost [10]. Previous research has demonstrated that earthworm digestion of sewage sludge can remove more of 90% of bacterial and fungal taxa, resulting in a significant reduction or, in some cases, the complete elimination of several pathogens [11,12]. Bacterial communities in sewage sludge are characterized as diverse and abundant [14], and these communities can exhibit significant variations between wastewater treatment plants (WWTPs) and across different countries [14,15]. However, it remains unclear whether the effects of earthworm gut transit on bacterial community composition and diversity and pathogen loads are general or if the outcomes mainly depend on the specific characteristics of the sewage sludge used.

To address these research questions, we used high-throughput 16S rRNA gene sequencing to analyse samples of sewage sludge from eight WWTPs immediately after collection and after transit through the earthworm gut. This approach enabled us to assess shifts in bacterial taxonomic and functional community composition, as well as alpha and beta diversity metrics and pathogen abundance. We hypothesized that, given the apparent elimination of numerous bacterial taxa during earthworm digestion, pathogenic bacteria would be either completely eradicated or substantially reduced in number.

## 2. Materials and Methods

### 2.1. Experimental Design and Sampling

We established eight earthworm boxes and fed them with fresh sewage sludge collected from eight wastewater treatment plants (WWTPs) located, respectively, in seven small villages and a dairy factory (InLeit Ingredients SLU, Teixeiro) in Galicia, Spain. The number of inhabitants in these localities were 37,761 (Vilagarcía de Arousa), 19,458 (Moaña), 12,589 (Ordes), 9834 (Caldas de Reis), 9580 (Burela), 6056 (Miño) and 5085 (Cerceda). A summary of sewage sludge characteristics is shown in Supplementary Materials Table S6. To collect fresh earthworm casts, we first washed mature earthworms in sequential baths of tap and sterile water. Then we put earthworms in sterile Petri dishes (five dishes with 20 worms per WWTP sewage sludge), which were incubated for one day to allow the earthworms defecate, that is release their casts (earthworm faeces) [10]. We collected the earthworm faeces under aseptic conditions storing them in sterile Eppendorf tubes at −80 °C.

### 2.2. Amplification, Sequencing and Analysis of 16S Region of rRNA Genes

We extracted DNA from sludge and earthworm cast samples (0.25 g fresh weight) using the MO-BIO PowerSoil^®^ kit (Qiagen, Madrid, Spain) without modifying the protocol in a laminar flow hood to avoid contamination. We amplified and sequenced V4 region of the 16S rRNA gene and process raw sequences with DADA2 (version 1.36.0) [16,17] following the protocol described in Domínguez et al. [10]. We classified ASVs using the RDP naive Bayesian classifier [18,19] against the SILVA database (version 138.2) and a bootstrap confidence level set at 80. We discarded ASVs unclassified at the phylum level. In total, 13,230 ASVs comprising 5,539,142 sequences were retained (55,391 ± 8370 sequences per sample, mean and standard deviation). Sequencing depth was optimal for our samples (Supplementary Materials Figure S1). We uploaded raw sequences to GeneBank SRA with accession number PRJNA1332974.

### 2.3. Bioinformatic and Statistical Analysis

We analyse and plot data with the following R packages: phyloseq (version 1.46.0) [20], tidyverse (version 2.0.0) [21], patchwork (version 1.3.2 ) [22], vegan (version 2.7.2) [23], ComplexHeatmap (version 2.18.0 ) [24], rstatix (version 0.7.3 ) [25], ggh4x (version 0.3.1 ) [26] and microeco (version 1.15.0) [27] and R version 4.4.0 [28]. We studied changes in bacterial phyla, genera and ASV abundance before and after earthworm gut digestion among WWTPs using DESeq2 package (version 1.42.1) [29]. This analysis was conducted on a prevalence-filtered dataset, thus removing 67% of bacterial ASVs, which represented 2.5% of the total sequences.

We defined native bacterial ASVs as those present in earthworm casts but absent from sewage sludge samples. After rarefying each dataset, we estimated the proportion of native ASVs for each WWTP individually. Native ASVs were considered those not shared by sludge and earthworm cast samples. We used generalized linear models (glm function, binomial family) to analyze the effect of the WWTP on the proportion of native ASVs, followed by Tukey post hoc tests (multcomp version 1.4-29 [30]). In addition, we assessed the effect of bacterial community composition of sewage sludge on native earthworm ASVs, by determining the number of shared ASVs across pairs, triples, quads, quintets, sextets, septets and octets of native ASVs from each WWTP. Low levels of shared taxa would indicate a predominant effect of sewage sludge on the composition of native ASVs, while high levels would indicate a minimal influence. We analysed the number of shared taxa across comparison levels (pairs to octets) using ANOVA and post hoc *t*-tests (*p*-values FDR-corrected) using the anova_test and *t*_test functions from the rstatix package.

We estimated taxonomic alpha-diversity as richness and inverse Simpson indexes. To calculate these indices, we rarefied the dataset 1000 times to estimate richness and diversity, and we then calculated the mean value for each index. To test the effect of earthworm digestion on alpha-diversity indices, we performed a two-way ANOVA including WWTP and sample type (sewage sludge vs. earthworm casts) as factors. This was followed by a paired *t*-test with corrected *p*-values (using the anova_test and *t*_test functions from the rstatix package). Taxonomic beta-diversity was measured as the difference in bacterial community composition (ASV level) in the sewage sludge and earthworm cast samples across WWTPs. We used distance matrices that either incorporated (Bray–Curtis) or did not incorporate (Jaccard) ASV abundance on the filtered ASV table (variance-stabilized transformation) and performed permutational multivariate analysis of variance (PERMANOVA) using the vegan package. We conducted pairwise PERMANOVA tests to look for differences before and after earthworm digestion across WWTPs. Finally, we used Principal Coordinate Analysis (PCoA) to represent modifications in beta diversity.

We predicted the functional composition of the bacterial communities using PICRUSt2 software package (version 2.6.2) [31]. Our samples had NSTI of 0.05 ± 0.02 (mean ± s.d.) similar to that well covered samples like those of human microbiome project, indicating that PICRUSt2 is expected to produce reliable results [31]. The effect of earthworm digestion on the alpha- and beta-diversity of functional composition was estimated in the same way as for the taxonomic composition. To facilitate comparisons, we conducted a detailed analysis of same metabolic pathways used by Domínguez et al. [10] and Gómez-Roel et al. [11] using the KEGGREST package (version 1.44.0) [32]. To test the effect of earthworm gut transit on the abundance of these metabolic pathways, we applied the same statistical models used to analyse changes in taxonomic and functional alpha-diversity.

To study the impact of earthworm gut transit on human bacterial pathogens (HBPs) we first assigned taxonomy at species level using the assignSpecies command from DADA2 (default settings). This procedure ensures unambiguous identifications, which is particularly suitable for 16S amplicon data [33]. Subsequently, the species-level taxonomic data were cross-referenced with the comprehensive catalogue of human bacterial pathogens (HBPs) compiled by Bartlett et al. [34], in order to determine the HBP species detected in both sewage sludge and earthworm casts. This list classifies the HPBs as established or putative depending on the number of registered infections and published articles [34].

### 2.4. Ethical Note

We followed the ASAB/ABS Guidelines for the Use of Animals in Research and complied with current Spanish regulation for the maintenance and usage of animals in scientific research (RD53/2013). Since we used an earthworm species as the experimental model, ethical committee approval was not required. Throughout the experiment, no earthworm exhibited adverse signs as a result of the experimental manipulations (i.e., being placed in a Petri dish to defecate).

## 3. Results

### 3.1. Impact of Earthworm Digestion on the Composition of Bacterial Communities in Sewage Sludge

The bacterial assemblages in sewage sludge from the eight wastewater treatment plants (WWTPs) were predominantly composed of bacteria in the phyla *Pseudomonadota*, *Actinomycetota*, *Bacteroidota* and *Chloroflexota*. However, the relative abundance of each phylum varied significantly across the different WWTPs (Table 1). Other bacterial phyla were also present in high relative abundance, but these were specific to certain WWTPs. For instance, *Bacillota* was abundant in the Burela and Vilagarcía de Arousa WWTPs, while *Acidobacteriota* was more prevalent in the Cerceda and InLeit WWTPs (Table 1). Additionally, several other bacterial phyla were present in the sewage sludge at lower relative abundance, and the particular phyla that were present strongly depended on the WWTP. This applied to *Verrucomicrobiota*, *Planctomycetota*, *Patescibacteria* and *Campylobacterota*, among others (Supplementary Materials Table S1).

Earthworm gut transit significantly influenced the abundance of major bacterial phyla in sewage sludge. In some cases, it consistently reduced the abundance of *Acidobacteroidota*, *Campylobacterota* and *Nitrospirota*, while it increased the abundance of *Actinomycetota* and *Planctomycetota* across most WWTPs (Supplementary Materials Table S2). In other instances, the effect varied depending on the WWTP, as observed for *Pseudomonadota*, *Bacillota*, *Bacteroidota*, *Chloroflexota* and *Verrucomicrobiota* (Supplementary Materials Table S2).

**Figure 1:**
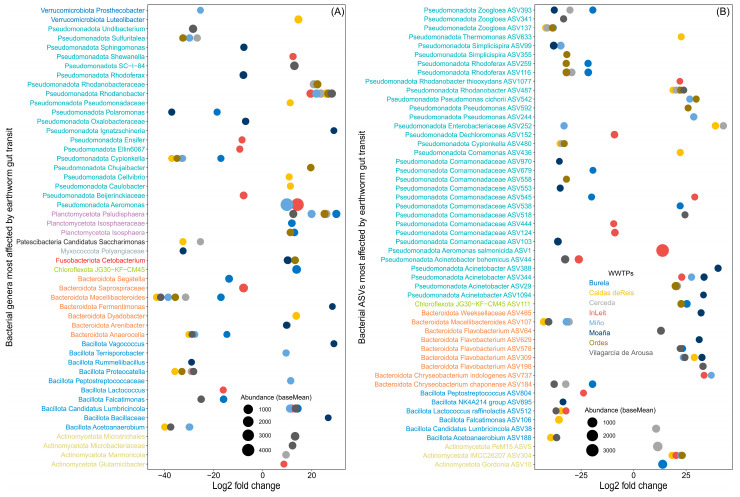
The fate of bacterial genera and ASVs in sewage sludge from different WWTPs after earthworm gut transit. DESeq2 showing the effect of earthworm gut transit on bacterial genera (**A**) and (**B**) ASVs of sewage sludge from eight WWTPs. Negative and positive log_2_fold changes indicate significant decreased or increased abundance (Wald test, *p* < 0.05) after earthworm gut transit (only the top six bacterial genera and ASVs per effect and WWTP are shown). The size of the points is proportional to the cumulative base mean value for bacterial ASVs (the mean of size-factor–normalized counts across all sample).

The variable effects of earthworm gut transit on bacterial phyla extend to the genus and ASV levels. Specifically, earthworm gut transit led to a substantial decrease (log_2_fold change < −10) in the abundance of bacterial genera such as *Macellibacteroides*, *Acetoanaerobium*, *Proteocatella* and *Cypionkella*, among others (Figure 1A, Supplementary Materials Table S3). By contrast, it caused a large increase (log_2_fold change > 10) in genera such as *Paludisphaera*, *Rhodanobacter* and *Aeromonas* (Figure 1A, Supplementary Materials Table S3). In some genera, the effect of earthworm gut transit was consistent across WWTPs, with increased abundance of genera like *Aeromonas*, *Gordonia*, *Microbacterium* and *Flavobacterium* and decreased abundance of *JGI 0001001-H03*, *Bacteroides*, *Acidovorax* and *Comamonas* (Figure 1A, Supplementary Materials Table S3). However, the abundance of other genera varied depending on the WWTP considered (Figure 1A, Supplementary Materials Table S3).

At the bacterial ASV level, the genera that underwent the greatest decreases after earthworm gut transit included *Zoogloea* (ASV393, ASV137, and ASV341), *Simplicispira* (ASV99 and ASV355), *Rhodoferax* (ASV116 and ASV259) and *Macellibacteroides* (ASV107) (Figure 1B, Supplementary Materials Table S4). By contrast, the ASVs that underwent the largest increases after gut transit were predominantly found in *IMCC26207* (ASV304), *Flavobacterium* (ASV198, ASV309, ASV578, ASV629 and ASV64), *Acinetobacter* (ASV1094, ASV29, ASV344, ASV388 and ASV44) and *Pseudomonas* (ASV244, ASV592 and ASV542) (Figure 1B, Supplementary Materials Table S4).

**Figure 2:**
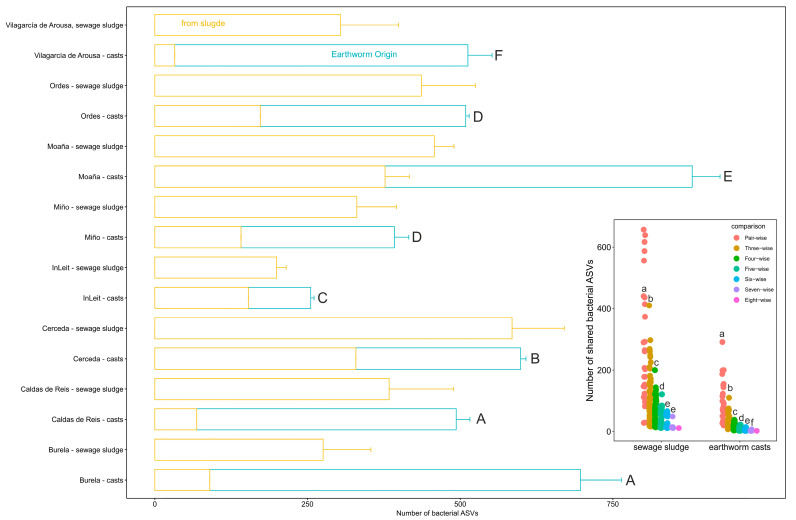
Source of bacterial taxa in earthworm casts. Bacteria are classified as “earthworm origin” if they appear only in earthworm casts. The inset plot displays the number of bacterial taxa of earthworm origin shared by earthworm casts of different WWTPs in pairs, triples, quad, quintet, sextet, septet and octet comparisons, as well as for the sewage sludge from all WWTPs. Different letters in the main plot and inset plots indicate significative differences (*p* < 0.05) in the amount of bacterial taxa among earthworm casts and in the number of shared bacterial taxa among comparisons respectively (Tukey HSD test).

Bacterial communities in earthworm casts from different WWTPs displayed varying proportions of native ASVs, i.e., those present in the cast samples but absent from the corresponding sewage sludge samples (GLM, χ^2^_7_ = 3795.8, *p* < 0.0001; Figure 2). Earthworm casts from the Vilagarcía de Arousa WWTP contained the highest proportion of native ASVs (93%), followed by casts from Burela and Caldas de Reis (~86%), Miño and Ordes (~65%), Moaña (58%), Cerceda (45%) and InLeit WWTPs (40%; Figure 2). Additionally, the number of shared bacterial ASVs among earthworm casts derived from different sewage sludge samples decreased significantly (ANOVA, F_6,268_ = 63, *p* < 0.0001) as the number of WWTPs included in the comparisons increased, from pairwise to eight-way (Figure 2 inset). This suggests that the source of the sewage sludge had a marked effect on the composition of native bacterial ASVs (Figure 2 insert).

### 3.2. Effect of Earthworm Digestion on Predicted Functional Composition of Bacterial Assemblages in Sewage Sludge

**Figure 3:**
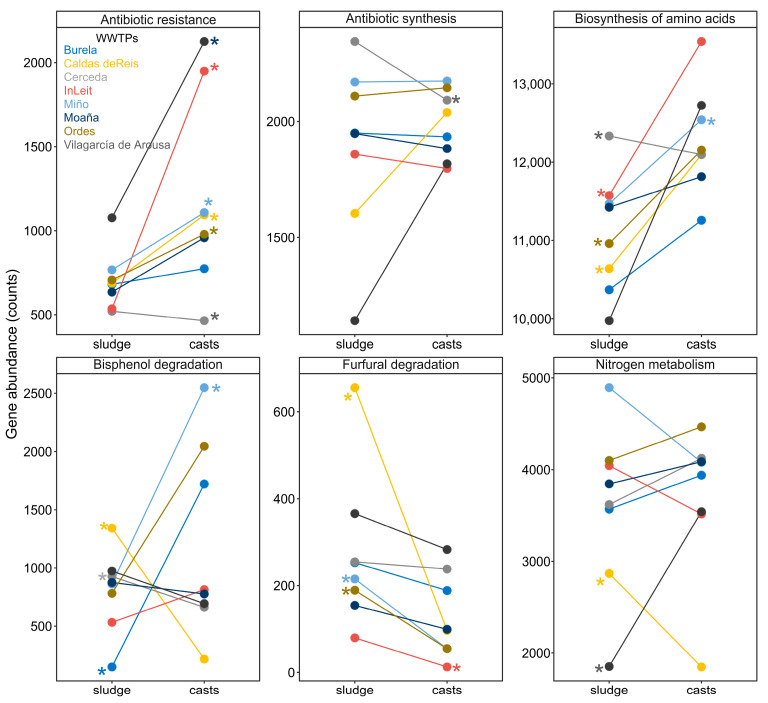
Gene abundance for selected functional pathways from bacterial communities in sewage sludge before (sewage sludge) and after transit through the gut of earthworms (casts) in different wastewater treatment plants (WWTPs). Significant differences (*p* < 0.05) in post hoc test (*t*-test) are shown with asterisks.

Earthworm gut transit significantly altered the abundance of genes across all functional pathways analysed (sample type effect, *p* < 0.01 for all significant pathways), except for those related to nitrogen metabolism, antibiotic synthesis and plant hormone synthesis (Figure 3). Additionally, the effect was significantly influenced by the WWTP for all metabolic pathways, except for antibiotic synthesis and plant hormone synthesis (interaction WWTP × sample type, *p* < 0.0001; Figure 3). The variation was mainly due to the fact that, for these metabolic pathways, earthworm gut transit did not consistently increase or decrease gene abundance across the different WWTPs (Figure 3). Earthworm gut transit generally increased the content of antibiotic resistance genes in most sewage sludge samples, except for those from the Vilagarcía de Arousa WWTP (Figure 3). A similar trend was observed for genes involved in amino acid biosynthesis. However, the effect on genes related to the degradation of bisphenol and furfural varied depending on the sewage sludge, with some showing an increase and others a decrease in gene content (Figure 3). By contrast, earthworm gut transit had a minimal impact on the gene content associated with antibiotic synthesis and nitrogen metabolism (Figure 3).

### 3.3. Impact of Earthworm Digestion on the Taxonomic and Functional Diversity of Bacterial Communities in Sewage Sludge

**Figure 4:**
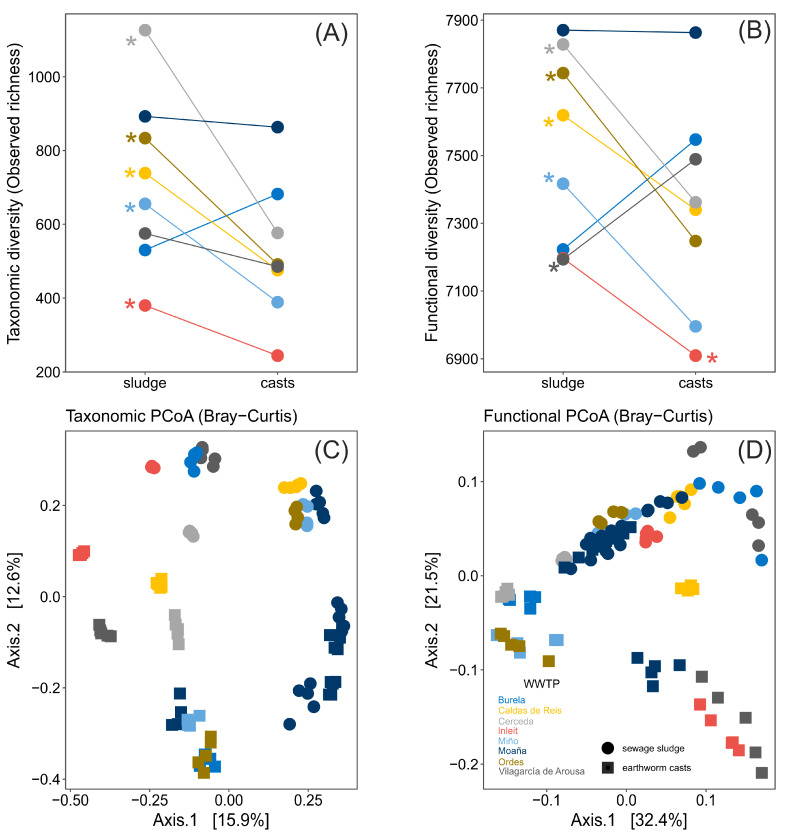
Modifications in taxonomic and functional diversity of bacterial communities of sewage sludge from several WWTPs before and after earthworm digestion showed as (**A**,**B**) taxonomic and functional alpha diversity richness and (**C**,**D**) taxonomic and functional beta diversity determined by PCoAs. Significant differences (*p* < 0.05) in post hoc test (*t*-test) are shown with asterisks in panels (**A**,**B**).

Earthworm digestion significantly altered the taxonomic and functional alpha-diversity (richness and diversity) of bacterial communities in sewage sludge, with the effect varying depending on the WWTP. Thus, there was a significant interaction WWTP × sample type for observed taxonomic richness (ANOVA, F_7,84_ = 6.41, *p* < 0.0001) and diversity (ANOVA, F_7,84_ = 4.85, *p* < 0.001), as well as for functional richness (ANOVA, F_7,84_ = 8.59, *p* < 0.0001) and diversity (ANOVA, F_7,84_ = 28.29, *p* < 0.0001, Figure 4A,B, Supplementary Materials Figure S2A,B). Specifically, in some WWTPs, gut transit increased taxonomic and functional alpha-diversity indices, whereas in others, it either decreased or had no effect (Figure 4A,B, Supplementary Materials Figure S2A,B).

Earthworm gut transit significantly altered the taxonomic and functional beta-diversity of bacterial communities in sewage sludge, with the changes being significantly dependent on the WWTP. Thus, there was a significant interaction WWTP × sample type for taxonomic beta-diversity measured with Bray–Curtis (PERMANOVA, F_7,84_ = 4.995, *p* = 0.001) and Jaccard indexes (PERMANOVA, F_7,84_ = 6.75, *p* = 0.001), as well as for functional beta-diversity measured with Bray–Curtis (PERMANOVA, F_7,84_ = 19.73, *p* < 0.0001) and Jaccard indexes (PERMANOVA, F_7,84_ = 9.79, *p* < 0.0001, Figure 4C,D, Supplementary Materials Figure S2C,D). Specifically, bacterial communities in sewage sludge grouped according to WWTP, for both taxonomic and functional beta-diversity measurements, and after earthworm gut transit, differed significantly from those in non-ingested sewage sludge (all pairwise comparisons between sewage sludge and earthworm casts, both within and across WWTPs, were significant, *p* < 0.05 for all, Supplementary Materials Table S6).

### 3.4. Effect of Earthworm Digestion on Human Bacterial Pathogens (HBPs)

**Figure 5:**
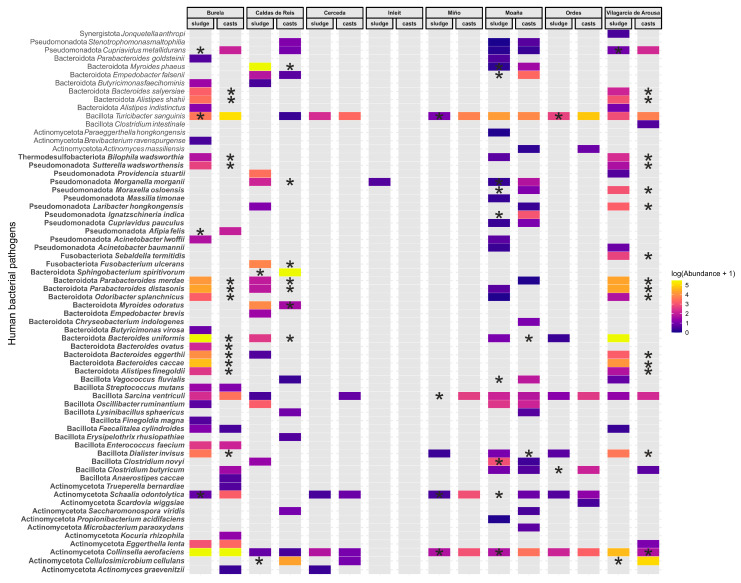
The fate of human bacterial pathogens (HBPs) in sewage sludge from different wastewater treatment plants (WWTPs) following earthworm gut transit. We classified HBPs as either established (bold font) or putative (plain font), based on the number of reported infections and published studies, as described by Bartlett et al. [34]. The location of asterisks indicates statistically significant increases (sewage sludge) or decreases (earthworm casts) after Wald tests (*p* < 0.05) in abundance within each WWTP (Wald test).

We identified 66 human bacterial pathogens (HBPs) in the dataset, although the overall abundance was relatively low, accounting for only 29,543 sequences from a total of 5,539,142 (Figure 5). Burela, Caldas de Reis, Moaña and Vilagarcía de Arousa WWTPs exhibited the highest diversity and abundance of HBPs. By contrast, sludge samples from the Cerceda, Miño and Ordes WWTPs and from the InLeit WWTP showed the lowest levels, with the latter containing almost no detectable HBPs (Figure 5). Transit through the earthworm gut generally reduced or eliminated many HBPs, including *Alistipes finegoldii*, *A. shahii* and several species of *Bacteroides* and *Parabacteroides* (Figure 5, Supplementary Materials Table S5). For other HBPs, the effect of earthworm gut transit (i.e., an increase or decrease in abundance) varied depending on the WWTP of origin. This was observed for *Collinsella aerofaciens*, *Myroides odoratus* and *Moraxella osloensis* (Figure 5, Supplementary Materials Table S5). A subset of HBPs consistently increased in abundance after earthworm gut transit, including *Cellulosimicrobium cellulans*, *Cupriavidus metallidurans*, *Schaalia odontolytica* and *Turicibacter sanguinis* (Figure 5, Supplementary Materials Table S5).

## 4. Discussion

Bacterial communities in sewage sludge differed markedly among wastewater treatment plants (WWTPs) as expected [14,15]. However, the levels of diversity, while consistent with previous reports [10,11], were much lower than those reported in recent global surveys [14,15]. We found that the earthworm digestion significantly altered both the composition and diversity of bacterial communities across the eight WWTPs studied, mirroring patterns previously observed in fungal communities [10,11,12]. The bacterial communities present in earthworm casts were predominantly composed of native taxa, a finding that is consistent with earlier observations from research on sewage sludge [10,11] and also on earthworms feeding on soil [35]. Although the proportion of native bacteria was generally high in the present study, it varied considerably among WWTPs, suggesting that the initial bacterial composition of the sewage sludge may play an important role. This interpretation is further supported by the limited overlap in the composition of native bacterial communities observed in the earthworm casts. An alternative explanation could involve the physicochemical and microbiological characteristics of the sewage sludge; however, these parameters were relatively consistent across WWTPs. The factors driving both the proportion and composition of native bacteria therefore remain unresolved and warrant further investigation.

Regarding bacterial abundance, we found that the effect of earthworm digestion varied substantially among bacterial taxa and across WWTPs. For example, some genera, such as *Acetoanaerobium*, *Macellibacteroides* and *Cypionkella,* were consistently suppressed following passage through the earthworm gut, whereas others, including *Rhodanobacter* and *Paludisphaera*, were markedly enriched. These shifts in abundance also had a significant impact on bacterial alpha-diversity, an effect that was strongly influenced by the specific WWTP, as previously reported for fungal communities [12]. Changes in both bacterial composition and alpha-diversity led to significant alterations in beta-diversity, which were again largely dependent on the WWTP consistent with earlier findings on fungal communities [12]. These results further support the hypothesis that the initial bacterial composition of sewage sludge may play a key role in shaping the bacterial communities present in earthworm casts.

Consistent with the observed modifications in bacterial composition and diversity, earthworm digestion also markedly altered the composition and diversity of predicted functional profiles of bacterial communities, as inferred using PICRUSt2. The observed increases or decreases in the gene content of various metabolic pathways were largely dependent on the specific WWTP, as previously reported [11]. Notably, in most WWTPs, earthworm gut transit led to a significant increase in gene content associated with antibiotic resistance. This finding contrasts with those of previous studies suggesting that vermicomposting may reduce the abundance of antibiotic resistance genes [13,36].

Finally, we found that the human bacterial pathogen (HBP) load was generally low in sewage sludge and varied greatly across samples. This may be due to the fact that sewage sludge from small urban WWTPs typically contains lower concentrations of pathogens than sewage sludge from larger cities [37]. It has been established that vermicomposting can reduce or eliminate HBPs from sewage sludge [12,38,39], and we indeed observed that passage through the earthworm gut tended to reduce or eliminate most of the HBPs detected. Unexpectedly, however, the abundance of certain HBPs increased after passage through the gut. This particularly applied to taxa such as *Klebsiella pneumoniae*, *Morganella morganii* and *Enterobacter cloacae*, and the pattern has also been reported for other bacterial pathogens and earthworm species fed different sources [40]. This finding raises concerns, as it suggests that earthworms could act as vectors of HBP transmission, potentially facilitating the survival or proliferation of specific HBPs during vermicomposting. Further research is required to determine the prevalence and viability of these pathogens in the final vermicompost product, as previously reported [41]. One possible explanation for the increased abundance of some HBPs may be the physiological stress experienced by earthworms by living in and feeding on sewage sludge, as elevated stress levels are associated with weakened immune responses [42]. However, although stress on earthworms can be exacerbated by pollutants commonly present in sewage sludge, such as pesticides, pharmaceuticals and microplastics [43,44,45], we did not observe a consistent increase in HBP abundance in earthworm casts from any specific WWTP. This suggests that additional factors may influence the selective enrichment of specific HBPs. One such factor is the presence of anaerobic niches that can promote the proliferation of certain microbial populations [46]. Moreover, shifts from aerobic to anaerobic conditions may steer bacterial communities toward anaerobic or facultative genera, such as *Clostridium sensu stricto* [47]. These low-oxygen microenvironments within earthworm guts could facilitate the enrichment of pathogenic species and enhance opportunities for gene exchange [48]. Finally, the digestive processes of earthworms themselves may play a role in modulating horizontal gene transfer and the accumulation of mobile genetic elements [49] favouring the emergence and/or resistance of some HBPs.

## 5. Conclusions

The study findings showed that bacterial communities in sewage sludge differed substantially before and after passage through the earthworm gut. The shift was primarily driven by the dominance of native bacterial taxa in earthworm casts that were absent from the original sludge. Consequently, the significant alterations in both alpha- and beta-diversity metrics were strongly dependent on the origin of the sewage sludge. The taxonomic changes were mirrored by corresponding shifts in predicted functional profiles, which were likewise influenced by the specific sewage sludge source. These findings highlight the need for further research to elucidate the extent to which the characteristics of sewage sludge impact the taxonomic composition and functional potential of bacterial communities following transit through the earthworm gut. The sewage sludge samples analysed in this study exhibited low levels of HBPs, most of which were reduced following gut transit. However, the abundance of some HBPs increased after gut transit, raising the question as to whether these HBPs persist in the final vermicompost. To better quantify the changes (whether reduced or increased HBP abundance), future studies should include analysis of vermicompost and apply quantitative methods such as qPCR, which yield more accurate assessment, to complement amplicon-based approaches.

## Figures and Tables

**Table 1:** Abundance of main bacterial phyla of sewage sludge before (sewage sludge) and after transit through the gut of earthworms (casts). Mean and standard errors of relative abundances.

Phylum	Sample Type	Burela	Caldas de Reis	Cerceda	Inleit	Miño	Moaña	Ordes	Vilagarcia de Arousa
*Acidobacteriota*	sewage sludge	0.03 ± 0.02	0.09 ± 0.02	7.5 ± 0.9	16.2 ± 1.8	1.7 ± 0.4	1.1 ± 0.2	3.6 ± 0.3	0.14 ± 0.03
	casts	0.004 ± 0.002	0.009 ± 0.004	0.08 ± 0.01	0.06 ± 0.03	0.01 ± 0.01	0.4 ± 0.1	0.014 ± 0.01	0.26 ± 0.05
*Actinomycetota*	sewage sludge	6.2 ± 0.9	8.6 ± 1.1	30.8 ± 2.1	3.4 ± 0.3	20.1 ± 2.2	21.9 ± 1.8	18.1 ± 1.7	0.61 ± 0.09
	casts	54.6 ± 1.1	2.1 ± 0.3	66.5 ± 1.4	9.2 ± 0.5	69.1 ± 2.7	33.8 ± 1.9	71.6 ± 3.3	11.2 ± 1.02
*Bacillota*	sewage sludge	16.6 ± 3.4	2.6 ± 0.2	0.9 ± 0.1	0.31 ± 0.02	0.6 ± 0.1	5.4 ± 1.2	1.4 ± 0.1	10.2 ± 2.6
	casts	18.8 ± 0.7	0.5 ± 0.1	2.5 ± 0.2	0.8 ± 0.1	4.8 ± 0.4	3.1 ± 0.2	4.9 ± 0.6	4.3 ± 0.6
*Bacteroidota*	sewage sludge	15.6 ± 2.4	18.6 ± 1.1	1.2 ± 0.1	4.9 ± 0.3	7.4 ± 0.5	12.9 ± 1.4	13.9 ± 0.9	22.2 ± 5.8
	casts	2.3 ± 0.6	54.4 ± 1	0.7 ± 0.1	0.4 ± 0.1	1.5 ± 0.3	10.1 ± 0.4	2.8 ± 0.3	9.6 ± 0.9
*Campylobacterota*	sewage sludge	0.4 ± 0.1	4.2 ± 1.4	0.007 ± 0.004	0 ± 0	0.16 ± 0.02	0.14 ± 0.05	0.22 ± 0.07	2.4 ± 0.9
	casts	0.001 ± 0.001	0. 2 ± 0.1	0 ± 0	0.001 ± 0.001	0.001 ± 0.001	0.3 ± 0.1	0.001 ± 0.001	0 ± 0
*Chloroflexota*	sewage sludge	0.002 ± 0.001	0.7 ± 0.2	17.9 ± 1.2	7.9 ± 0.7	19.6 ± 0.3	5.9 ± 0.7	17.5 ± 1.2	0.006 ± 0.003
	casts	3.6 ± 0.3	0.04 ± 0.01	7.9 ± 0.8	10.2 ± 0.8	5.4 ± 0.5	2.9 ± 0.3	2.9 ± 0.2	0.3 ± 0.1
*Nitrospirota*	sewage sludge	0 ± 0	0 ± 0	1.4 ± 0.1	0.02 ± 0.01	2.1 ± 0.2	0.06 ± 0.02	1.2 ± 0.1	0 ± 0
	casts	0.002 ± 0.002	0 ± 0	0 ± 0	0 ± 0	0 ± 0	0.014 ± 0.004	0.001 ± 0.001	0.02 ± 0.01
*Planctomycetota*	sewage sludge	0.001 ± 0.001	0.4 ± 0.1	7.5 ± 0.8	4.1 ± 0.3	0.6 ± 0.2	2.1 ± 0.5	1.2 ± 0.1	0.004 ± 0.002
	casts	6.3 ± 1.2	0.5 ± 0.1	4.5 ± 0.5	2.9 ± 0.5	0.9 ± 0.1	1.6 ± 0.4	2.6 ± 0.35	6.6 ± 1.5
*Pseudomonadota*	sewage sludge	60.7 ± 6.4	61.1 ± 0.7	24.8 ± 1.2	62.1 ± 1.9	41.1 ± 2. 1	45.1 ± 2.4	35.4 ± 0.6	62.8 ± 9.1
	casts	13.16 ± 1.76	33.7 ± 1.5	13.2 ± 0.6	74.6 ± 1.6	16.7 ± 2.9	43.9 ± 1.2	11.9 ± 0.8	65.5 ± 3.1
*Verrucomicrobiota*	sewage sludge	0.14 ± 0.02	0.16 ± 0.01	3.7 ± 0.6	0.15 ± 0.02	0.6 ± 0.2	0.7 ± 0.2	0.71 ± 0.1	0.03 ± 0.01
	casts	0.2 ± 0.1	7.1 ± 1.5	3.6 ± 0.6	0.03 ± 0.00	0.03 ± 0.01	0.5 ± 0.1	0.03 ± 0.01	1.2 ± 0.4

## Data Availability

Sequences were uploaded to the GenBank SRA database under accession PRJNA1332974.

## Supplementary Materials

The following supporting information can be downloaded at: https://www.mdpi.com/article/10.3390/microorganisms13112507/s1, Figure S1: Rarefaction curves indicating the number of amplicon sequence variants (ASVs) found in each sample before (sewage sludge) and after (casts) earthworm gut transit in eight different wastewater treatment plants; Figure S2: Changes in taxonomic and functional diversity of bacterial communities of sewage sludge from several WWTPs before and after passage through the gut of the earthworm species Eisenia andrei are shown as (A,B) taxonomic and functional alpha diversity estimated as observed inverse Simpson index and (C,D) taxonomic and functional beta diversity determined by principal coordinate analysis of Jaccard distances. Asterisks in panel A and B indicate significant differences between sewage sludge and earthworm casts within each WWTP (paired *t*-test, FDR-corrected); Table S1: Relative abundance of bacterial phyla of sewage sludge from eigth WWTP before (sewage sludge) and after earthwrom gut transit (casts); Table S2: Differential abundance in bacterial phyla of sewage slugde from different WWTPs and casts of earthworm species *Eisenia andrei* analyzed using negative binomial models as implemented in the package DESeq2 [29]; Table S3: Differential abundance in bacterial genera of sewage slugde from different WWTPs and casts of earthworm species *Eisenia andrei* analyzed using negative binomial models as implemented in the package DESeq2 [29]; Table S4: Differential abundance in bacterial ASVs of sewage slugde from different WWTPs and casts of earthworm species *Eisenia andrei* analyzed using negative binomial models as implemented in the package DESeq2 [29]; Table S5: Differential abundance in human bacterial pathogens (HBPs) of sewage slugde from different WWTPs and casts of earthworm species *Eisenia andrei* analyzed using negative binomial models as implemented in the package DESeq2 [29]; Table S6: Main physico-chemical characteristics of sewage sludge from eigth WWTPs (Mean and standard errors, N = 5).
