# Supplementary material for: Effect of Earthworm Digestion on Abundance, Composition and Diversity of Bacterial Pathogens in Sewage Sludge from Wastewater Treatment Plants
Source: Microorganisms. 2025 Oct 31;13(11):2507. doi: 10.3390/microorganisms13112507 (PMC12654803; doi:10.3390/microorganisms13112507)
Supplement: Supplementary file 1 [file microorganisms-13-02507-s001.zip › Supplementary_Figures.pdf]

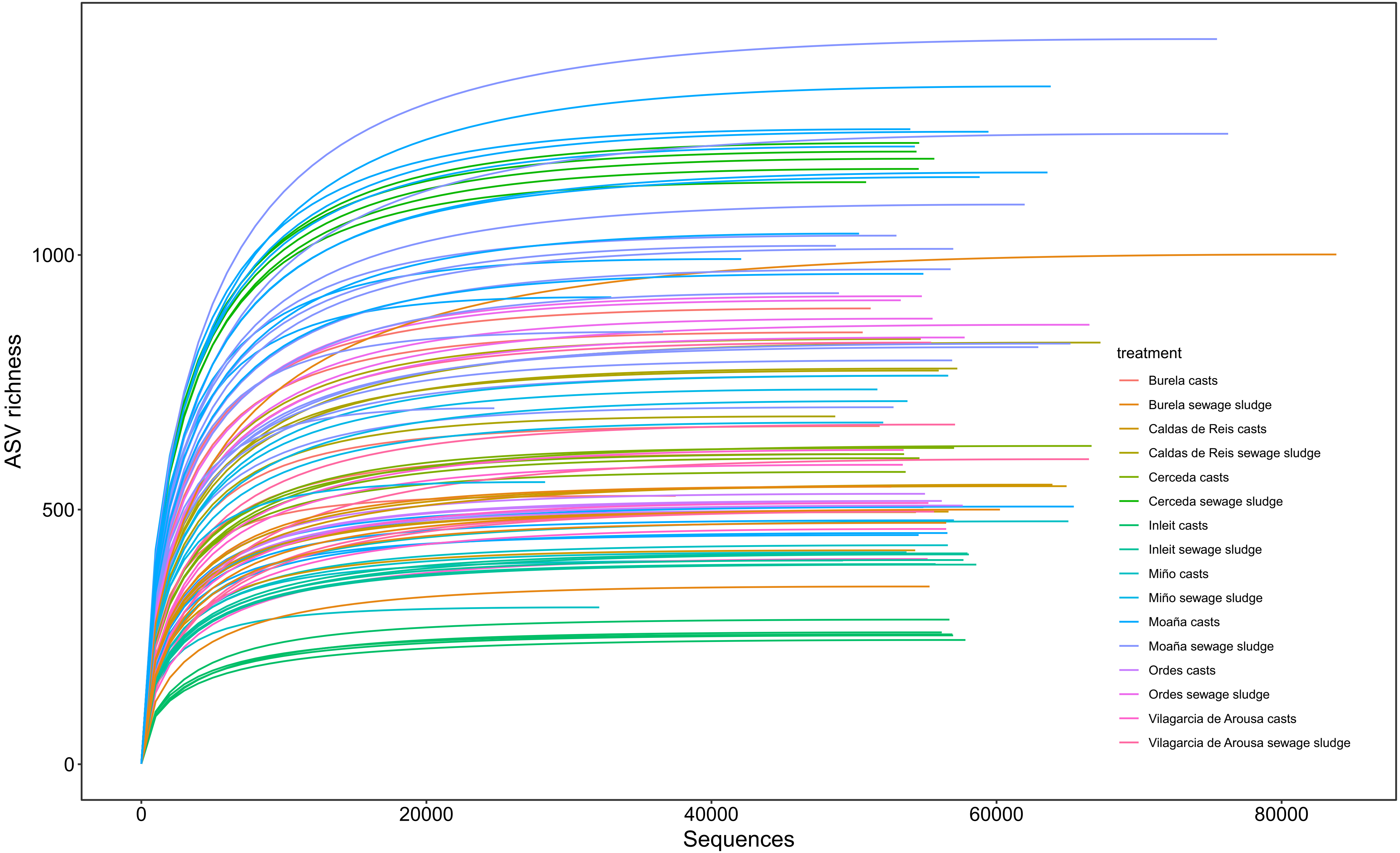

Supplementary Figure 1. Rarefaction curves indicating the number of amplicon sequence variants (ASVs) found in each sample before (sewage sludge) and after (casts) earthworm gut transit in eight different wastewater treatment plants.

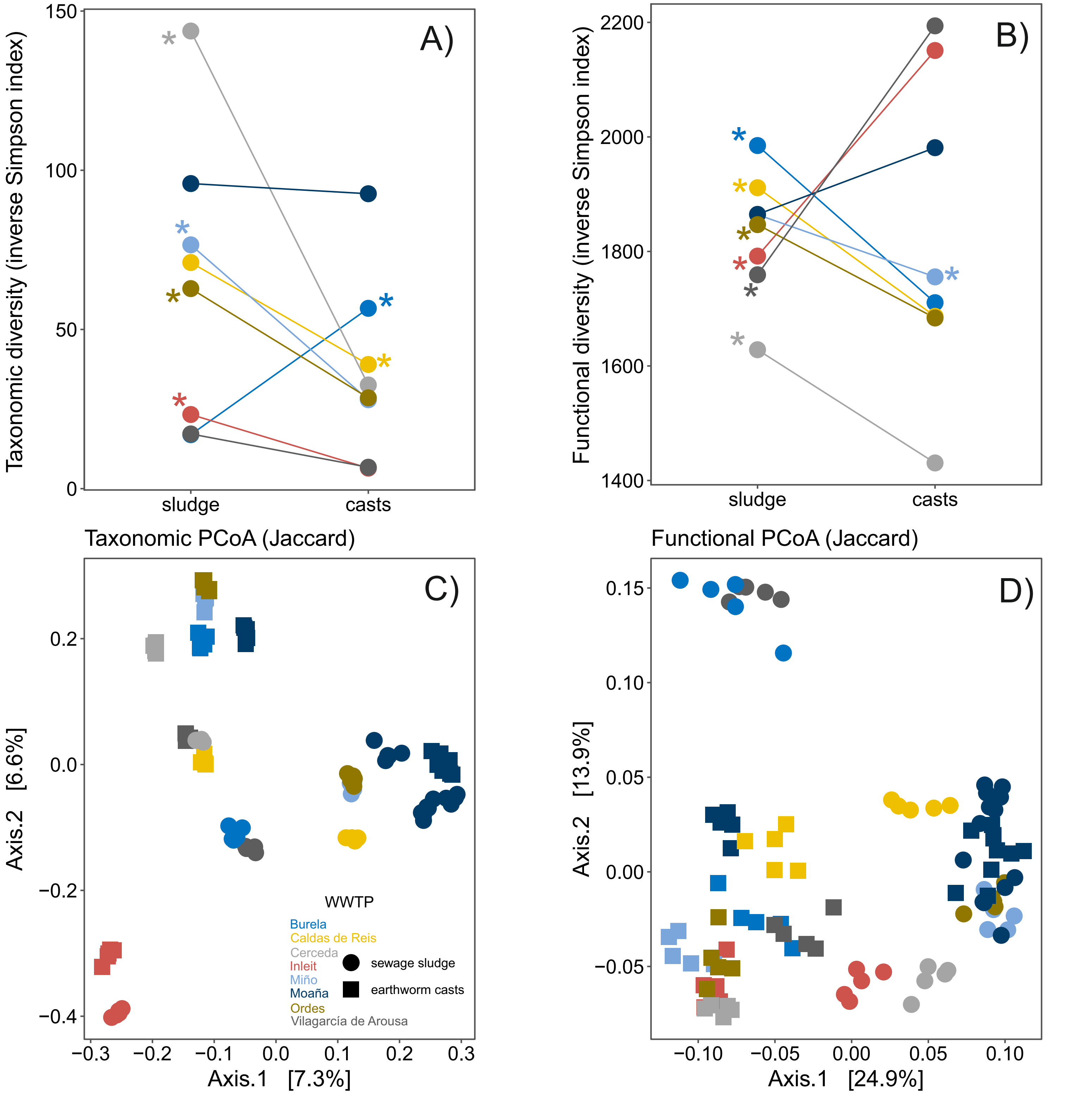

Supplementary Figure 2. Changes in taxonomic and functional diversity of bacterial communities of sewage sludge from several WWTPs before and after passage through the gut of the earthworm species *Eisenia andrei* are shown as (A,B) taxonomic and functional alpha diversity estimated as observed inverse Simpson index and (C,D) taxonomic and functional beta diversity determined by principal coordinate analysis of Jaccard distances. Asterisks in panel A and B indicate significant differences between sewage sludge and earthworm casts within each WWTP (paired t-test, FDR-corrected).
